# Analysis of serum reproductive hormones and ovarian genes in pubertal female goats

**DOI:** 10.1186/s13048-023-01150-0

**Published:** 2023-04-06

**Authors:** Yanyun Zhu, Jing Ye, Ping Qin, Xu Yan, Xinbao Gong, Xiaoqian Li, Ya Liu, Yunsheng Li, Tong Yu, Yunhai Zhang, Yinghui Ling, Juhua Wang, Hongguo Cao, Fugui Fang

**Affiliations:** 1 Department of Animal Veterinary Science, College of Animal Science and Technology, Anhui Agricultural University, 130 Changjiang West Road, Hefei, Anhui 230036 China; 2 Anhui Province Key Laboratory of Local Livestock and Poultry, Genetical Resource Conservation and Breeding, College of Animal Science and Technology, Anhui Agricultural University, Hefei, Anhui 230036 China

**Keywords:** Goats, Puberty, Reproductive hormones, Ovary, RNA-seq

## Abstract

**Background:**

Age at puberty is an important factor affecting goat fertility, with endocrine and genetic factors playing a crucial role in the onset of puberty. To better understand the relationship between endocrine and genetic factors and mechanisms underlying puberty onset in goats, reproductive hormone levels were analyzed by ELISA and ultraperformance liquid chromatography–multiple reaction monitoring–multistage/mass spectrometry and RNA sequencing was performed to analyze ovarian genes.

**Results:**

Serum follicle stimulating hormone, luteinizing hormone, estradiol, 11-deoxycortisol, 11-deoxycorticosterone, corticosterone, cortisone, and cortisol levels were found to be higher but progesterone were lower in pubertal goats as compared to those in prepubertal goats (*P* < 0.05). A total of 18,139 genes were identified in cDNA libraries, and 75 differentially expressed genes (DEGs) were identified (|log_2_ fold change|≥ 1, *P* ≤ 0.05), of which 32 were significantly up- and 43 were down-regulated in pubertal goats. Gene ontology enrichment analyses indicated that DEGs were mainly involved in “metabolic process,” “signaling,” “reproduction,” and “growth.” Further, DEGs were significantly enriched in 91 Kyoto Encyclopedia of Genes and Genomes pathways, including estrogen signaling pathway, steroid hormone biosynthesis, and cAMP signaling pathway. Bioinformatics analysis showed that *PRLR* and *THBS1* were highly expressed in pubertal ovaries, and *ZP3*, *ZP4*, and *ASTL* showed low expression, suggesting their involvement in follicular development and lutealization.

**Conclusions:**

To summarize, serum hormone changes and ovarian DEGs expression were investigated in our study. Further studies are warranted to comprehensively explore the functions of DEGs in goat puberty.

**Supplementary Information:**

The online version contains supplementary material available at 10.1186/s13048-023-01150-0.

## Introduction

In female animals, puberty is defined as the occurrence of the first estrus and ovulation [1], and this process affects their fertility. Animal experiences several estrous cycles before mating, their reproductive ability improves to a certain extent, implying that their reproductive performance is relatively better by early puberty [2]. Puberty and the initiation of normal estrous cycles involve a complex series of events. Puberty is influenced by diverse factors, such as the environment [3], nutrition [4], and endocrine [1, 5, 6] as well as genetic factors [7, 8]. It follows that the age at puberty has an important impact on the animals fertility [9] and an appropriate adjustment of the puberty onset age can provide new options to improve the litter rate.

The initiation of puberty is associated with the maturation of the hypothalamic–pituitary–ovarian (HPO) axis [10]. The ovary plays a pivotal role during puberty, directly mediating follicular maturation and gonadal steroid hormone secretion, thereby affecting reproductive performance [11]. With the onset of puberty, animals experience a change in their endocrine function; for instance, there are changes in the levels of hormones secreted by the pituitary and ovary [10, 12]. In Meishan gilts, luteinizing hormone (LH) and estradiol (E_2_) levels were observed to increase in the last 2 weeks in the prepubertal stage; further, the increase in LH levels preceded that of E_2_ levels [13]. In contrast, no significant changes were found in the levels of follicle stimulating hormone (FSH), but in the pubertal stage, the levels of LH, FSH, and progesterone (P_4_) were higher [13]. A pulsatile increase in LH, a more stable increase in FSH, and a significant increase in E_2_ levels have been previously reported before puberty in cattle [2], with P_4_ levels remaining relatively stable during the prepubertal stage and increasing during the pubertal stage [14]. Further, Prunier et al. [13] reported that cortisol appeared to inhibit LH secretion. Similar to E_2_ and P_4_, cortisol, which is secreted by the adrenal cortex, is a derivative of cholesterol and is a steroid hormone. The adrenal gland also plays a key role in puberty [15], particularly in regulating glucose metabolism, participating in immune regulation, and secreting certain sex hormones. Nevertheless, adrenal gland endocrine changes during puberty remain unclear, and only a few studies have explored changes in the levels of 11-deoxycortisol (11-Deo), 11-deoxycorticosterone (Doc), corticosterone (Cort), cortisone, or cortisol during puberty.

The mechanism underlying the initiation of puberty is complex; it is notable that hormone metabolism, follicular development, and ovarian function are regulated by a large number of genes [16]. Moreover, polygenic inheritance is closely associated with the transcription of receptor genes [8]. Zhao et al. [16] performed a transcriptome analysis of goat ovaries and differentially expressed genes (DEGs) were found to be largely associated with “cellular process”, “metabolic process”, and “biological regulation”, along with “reproduction” and “reproductive process”. Yang et al. [17] and Chu et al. [18] performed RNA-sequencing (RNA-seq) and identified DEGs in porcine ovaries. Their results indicated that “ECM-receptor interaction” and “cell adhesion molecules” pathways are essential for follicular development and that DEGs involved in these pathways were mostly downregulated during puberty. Yang et al. [17] found that the expression of *INHBA* was downregulated during puberty in sows, which increased FSH levels and promoted follicular development; in addition, *IGF2R* and *IGFBP3* were reported to play a crucial role in follicular growth and development. Chu et al. [18] reported that *VEGF* and *STAR* affect follicular development and steroid hormone synthesis in sheep; moreover, DEGs involved in steroid biosynthesis and ovarian steroidogenesis were mostly upregulated. Quan et al. [19] conducted a similar transcriptomic investigation to identify DEGs between pregnant and nonpregnant goat ovaries, enhancing our understanding of the complex molecular regulatory mechanisms occurring during the development of pregnancy and reproduction in goats. Collectively, these data imply that DEGs in ovaries are inextricably linked to whether the animal is in estrus and to hormone secretion.

Goats (*Capra hircus*), an important domestic and commercial animal in China, are a source of meat, high-quality wool, and other products; however, they have low fecundity, which poses a serious threat to the future of the goat industry [19]. Anhui white goats (AWGs) are widely used a goat model animal to investigate reproductive mechanisms owing to the characteristics of early puberty, high fertility, and small body size [9, 19]. As mentioned above, numerous factors influence the initiation of puberty in animals. Endocrine and genetic factors jointly affect the onset of puberty and regulate each other. But there are no study on the relationship between endocrine and genetic. Herein, to explore the association of reproductive hormones and ovarian genes with the onset of puberty, we investigated the serum levels of gonadotropins and steroid hormones and mRNA expression profiles of ovaries from pubertal and prepubertal goats. Our results may provide more detailed information about hormone secretion and gene expression at the onset of puberty in goats and may helpful for goat breeding and provide a new view for improving animal reproductive performance and treating reproductive diseases.

## Results

### Levels of reproductive hormones

**Fig. 1:**
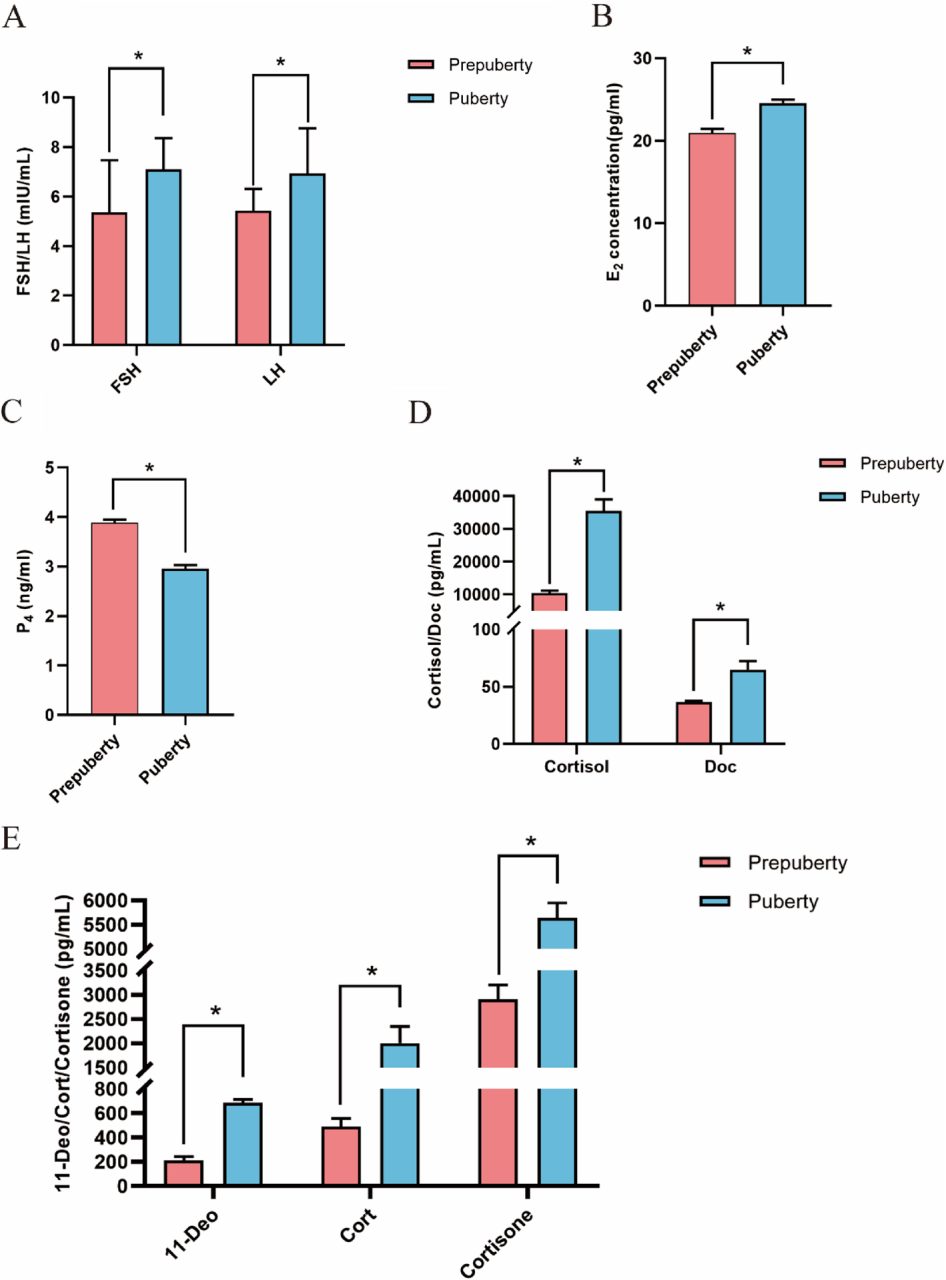
Serum FSH (**A**), LH (**A**), E_2_ (**B**), P_4_ (**C**), Cortisol (**D**), Doc (**D**), 11-Deo (**E**), Cort (**E**), and cortisone (**E**) levels in pubertal and prepubertal Anhui white goats. Values represent mean ± standard error. FSH, follicle stimulating hormone; LH, luteinizing hormone; E_2_, estradiol; P_4_, progesterone; Doc, 11-deoxycorticosterone; 11-Deo, 11-deoxycortisol; Cort, corticosterone

In comparison to prepubertal goats, pubertal goats showed a significant increase in the levels of serum FSH, LH (Fig. 1A), E_2_ (Fig. 1B), Doc, Cortisol (Fig. 1D), 11-Deo, Cort, and Cortisone (Fig. 1E) (*P* < 0.05). In contrast, serum P_4_ (Fig. 1C) levels were significantly decreased (*P* < 0.05) in pubertal goats.

### Identification and analysis of DEGs

**Fig. 2:**
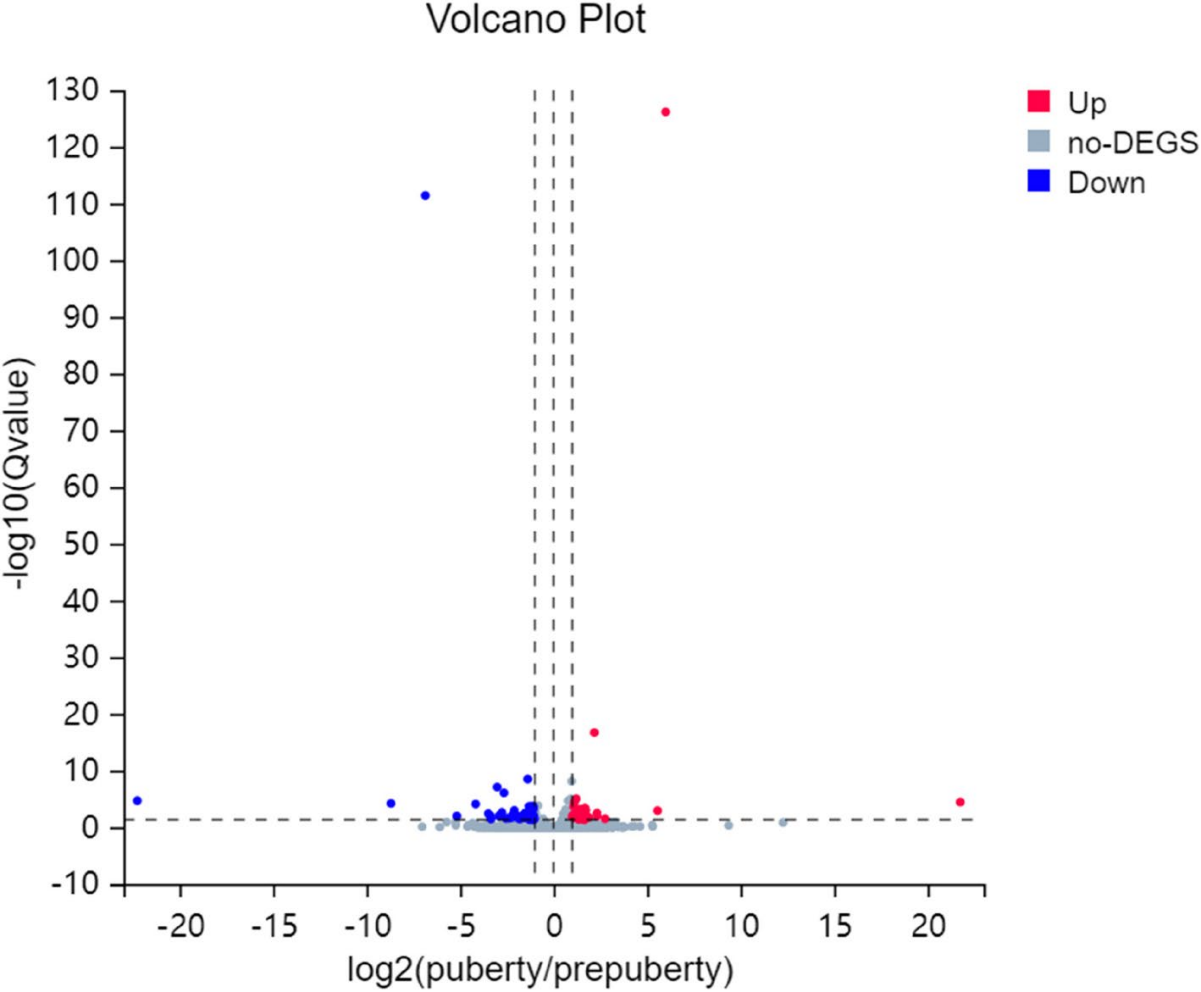
Volcano plots showing differentially expressed genes (DEGs) in puberty. Red represents relatively high expression, blue represents relatively low expression, and gray represents no change

In total, 18,139 genes were identified in the six cDNA libraries. Gene expression levels were evaluated using RSEM, and DESeq2 was used to analyze expression differences between prepubertal and pubertal goats. The information of gene alignment and genomic alignment was suppled in Table S1. The DEGs between prepuberty and puberty was visualised by volcano plots. Overall, 75 DEGs were identified of which 32 were up- and 43 were down-regulated (*Q*-value ≤ 0.05 and |log_2_ fold change (FC)|≥ 1.0) (Fig. 2 and Table S2). Cluster analyses of DEGs are presented in Fig. S1. Red and blue represent higher expression levels and lower expression levels, respectively. The results showed good reproducibility of data for each group of samples.

### Bioinformatics analysis of DEGs

#### Gene ontology (GO) analysis

**Fig. 3:**
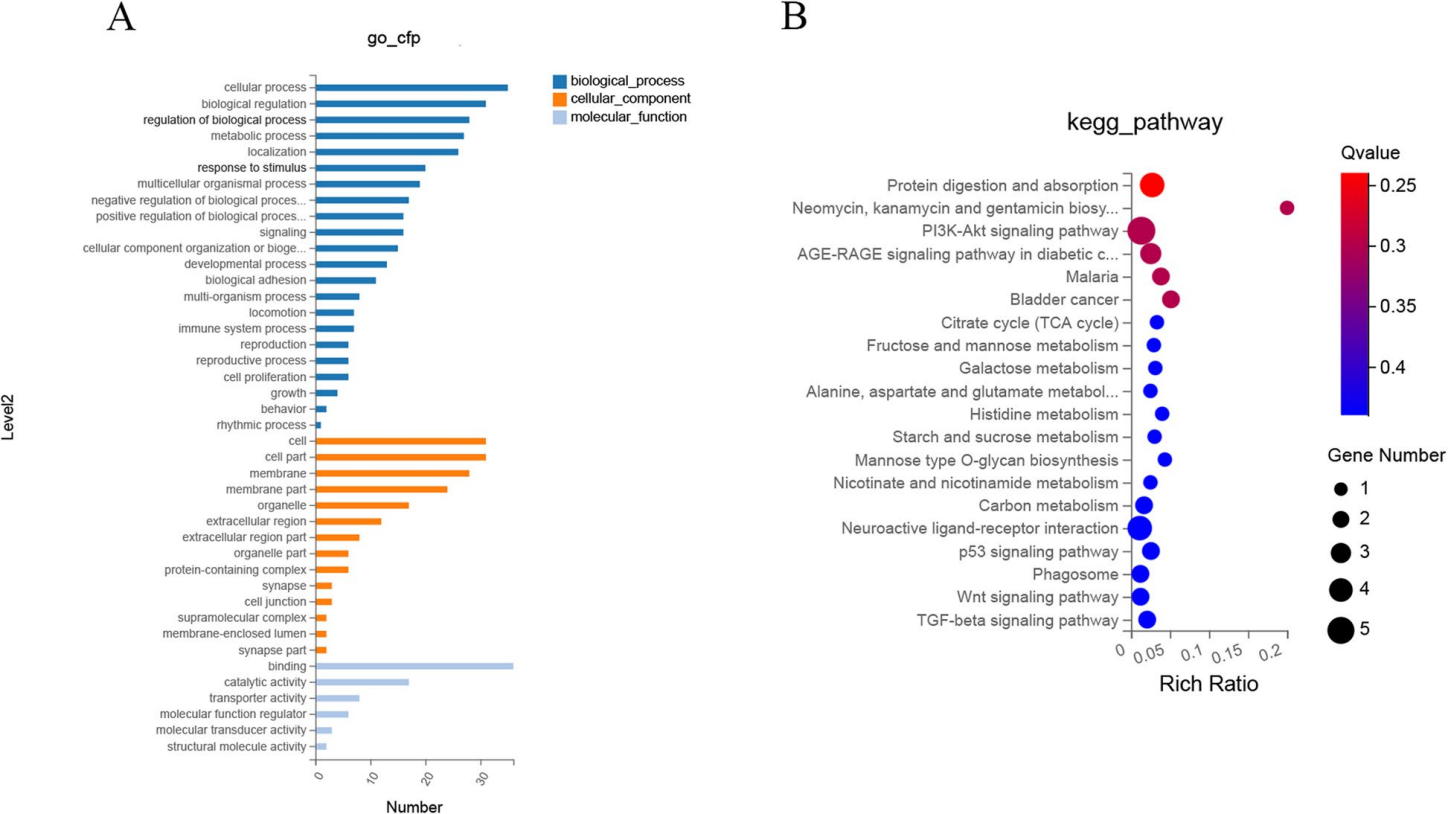
**A** Gene ontology analysis of differentially expressed genes. Genes are classified into the biological process, cellular component, and molecular function categories. **B** Top 20 enriched pathways. Colors from red to blue indicate *P* values from small to large. Bubble size indicates the number of differentially expressed genes in each biological process

To explore the functions of DEGs and the mechanisms underlying the onset of puberty, DEGs were annotated by GO analyses. In total, 42 GO terms were found to be significantly enriched (*P* ≤ 0.05). The results are shown in Fig. 3A and Table S3. In the biological processes (BP) category, the most abundantly annotated terms were “cellular process”, “biological”, “regulation of biological process”, and “metabolic process”. In the cellular components (CC) category, the most enriched terms were “cell”, “cell part”, “membrane”, and “membrane part”, and in the molecular functions (MF) category, the most abundant terms were “binding” and “catalytic activity”. Besides, many enriched GO terms in the BP category were associated with the onset of puberty, such as “metabolic process”, “signaling”, “reproduction”, and “growth”.

#### Kyoto Encyclopedia of Genes and Genomes (KEGG) pathway enrichment analysis

Figure 3B shows the top 20 enriched KEGG pathways. Overall, 75 DEGs were mapped to 91 KEGG pathways (Table S4), and three KEGG pathways (protein digestion and absorption; PI3K-Akt signaling pathway and p53 signaling pathway) which may relative to puberty onset were significantly enriched (*P* ≤ 0.05). In addition, numerous pathways were associated with puberty, such as the GnRH signaling pathway, estrogen signaling pathway, oxytocin signaling pathway, prolactin signaling pathway, steroid hormone biosynthesis, and cAMP signaling pathway.

#### Gene network analysis

**Fig. 4:**
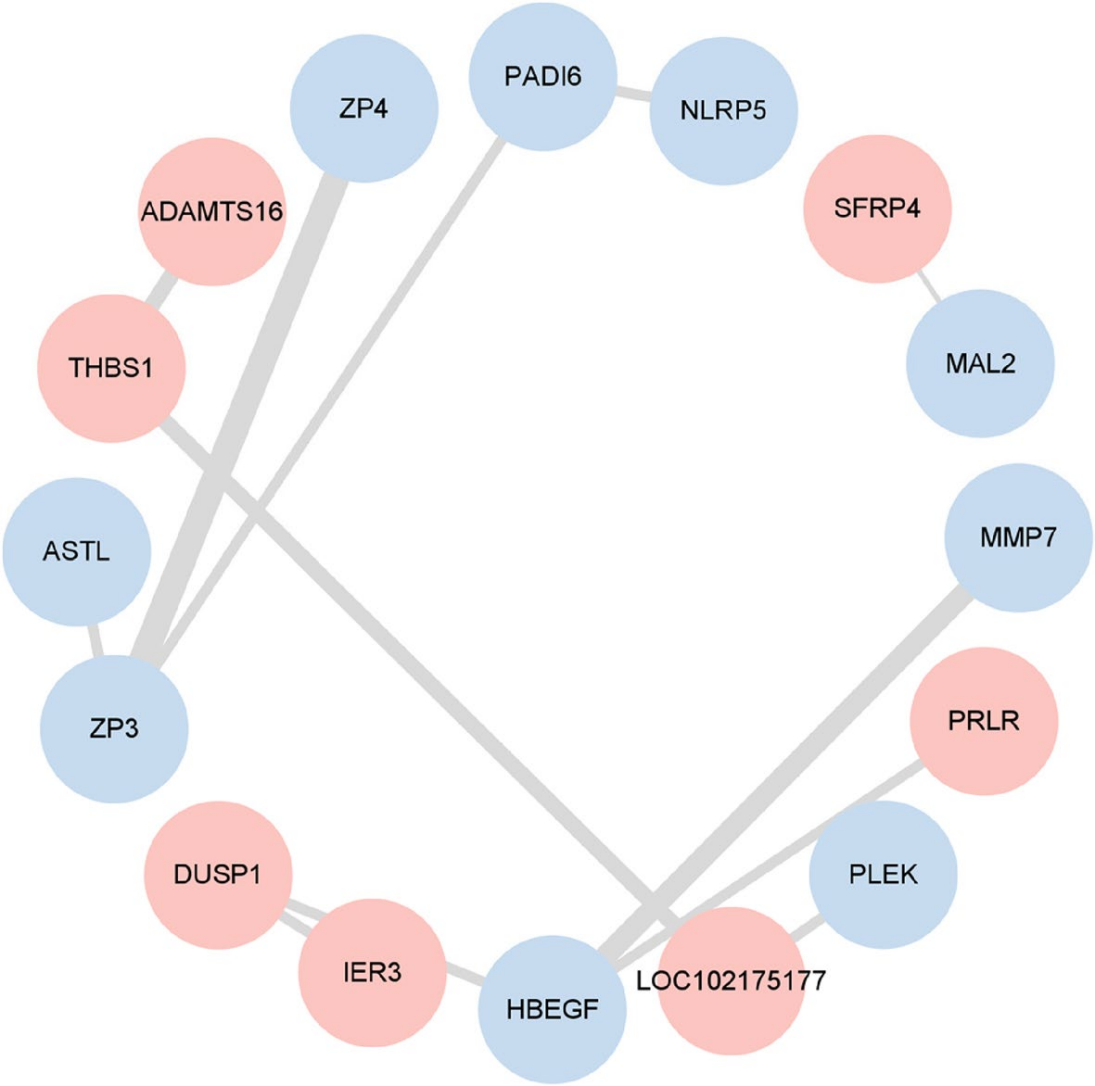
Gene network analyses of differentially expressed genes. The width of lines means indicates the type of interaction evidence. Red represents up- and blue represents downregulation

To identify key genes regulating puberty, a gene network was constructed with DEGs identified on evaluating prepubertal and pubertal ovaries. The gene network contained 16 nodes and 12 edges (interaction score > 0.4) (Fig. 4). The nodes represent proteins, the edges connecting two nodes imply a possible functional association between the two proteins, and the value of the edge width implies the amount of interaction type. The red nodes represent DEGs expressing up- during puberty, while blue represents down-regulation. The average node degree was 0.387. The hub genes *ZP3* (102183202), *HBEGF* (102185462), and *THBS1* (100860971) appeared to play a more important role in puberty. Moreover, other genes, such as *THBS1*, *PRLR* (100861318), and *DUSP1* (102180080), were highly expressed in pubertal ovaries, indicating that they are also vital for the initiation of puberty in goats.

### Real-time quantitative PCR (RT-qPCR) validation

**Fig. 5:**
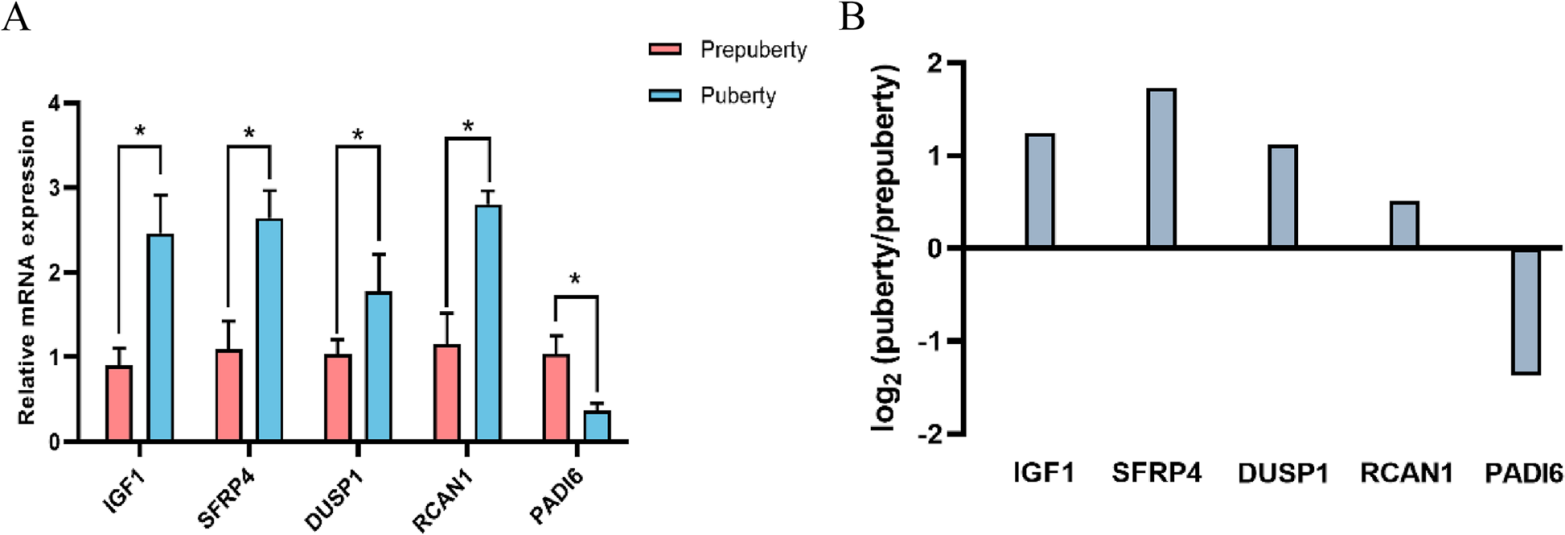
Validation of expression levels of five differentially expressed genes by RT-qPCR (**A**) and RNA-seq results of these genes (**B**)

Five genes were selected randomly and analyzed by RT-qPCR. In comparison with prepubertal AWGs, the expression levels of *IGF1*, *SFRP4*, *DUSP1*, and *RCAN1* were upregulated and those of *PADI6* were downregulated in pubertal AWGs (Fig. 5A). These expression patterns were consistent with RNA-seq data (Fig. 5B), indicating that RNA-seq results were reliable and could be used for mRNA differential expression analysis.

## Discussion

To investigate endocrine changes during puberty in goats, we herein determined LH, FSH, E_2_, P_4_, Cortisol, Doc, 11-Deo, Cort, and cortisone levels in prepubertal and pubertal goat serum by ELISA and UPLC-MRM-MS/MS. The onset of puberty begins with an increase in GnRH to activate the HPO axis. Many studies have reported that LH, FSH, and E_2_ levels show an incremental rise before puberty [20]. LH and FSH act together on the gonads to stimulate the secretion of sex steroid hormones during prepuberty, whereas high levels of E_2_ play a key role in the endocrine function of the hypothalamus and pituitary, suppressing the secretion of gonadotropins [21] and regulating follicular development in the ovary [22]. These theories have been previously verified in cattle (*Bos frontalis*) [14]: LH and FSH levels showed a simultaneously surge during prepuberty, while E_2_ levels increased after the surge in LH levels. In our study, we observed the increase of LH, FSH and E_2_ in puberty. The trend in all three was consistent with previous findings, and our detection of higher gonadotropins level in puberty may be due to missing the moment of their peak in prepuberty. Also, gonadotropins take time to metabolise, which may result in higher levels during puberty. The inhibitory effect of E_2_ on the hypothalamus and pituitary gland subsequently may attenuate the secretion of FSH and LH after 5 months of age. Moreover, P_4_, a steroid hormone secreted by the corpus luteum and placenta, also has an important role in puberty. Exogenous P_4_ has been shown to increase the LH secretion in prepuberty and to induce puberty initiation [23]. The stimulation of LH, FSH, and E_2_ at prepuberty leads to the luteinization of ovaries and secretion of P_4_ [24]. Luteinization of non-ovulatory ovaries still occurs in sheep prepuberty resulting in elevated P_4_ levels [25]. The higher P_4_ levels during puberty observed in our results may be due to the fact that P_4_ and E_2_ are synergistically stimulating follicle development and promoting ovulation at this time. After follicular discharge, luteinization of the ovary causes a surge in P_4_ levels and the phenomenon was found after estrus [14]. The increase in P_4_ levels may occur after 5 months of age. In general, no single reproductive hormone influences the course of puberty alone; they interact with each other to regulate the onset of puberty.

During E_2_ and P_4_ production, other steroid hormones, such as Cort and Cortisol, are also secreted. The adrenal cortex is the main source of estrogen and P_4_ during puberty; however, puberty is still evidently initiated even in the absence of the adrenal glands in animals [26]. Furthermore, in Sprague–Dawley rats, adrenalectomy at weaning age was found to result in the same delay in vaginal opening and ovulation [27]. The adrenal glands secrete some of gonadal hormones and a large amount of corticosteroids during puberty. We observed that the levels of serum 11-Deo, Cort, cortisol, and cortisone were significantly higher in pubertal goats than in prepubertal goats. Serum Doc concentrations were non-significantly increased during puberty. The increase in the levels of these hormones during puberty may be related to P_4_ secretion. High levels of corticosteroids promote ovulation and affect gonadotropin synthesis and LH peak at the first ovulation [26, 28]. According to our results, the increase in corticosteroid levels coincided with a surge in LH levels, but the increase in cortisol levels was contrary to the results reported by Prunier et al. [13]. These results imply differences in hormone secretion patterns between species. It is possible that there is some association between changes in corticosteroid and LH levels during puberty, regulating the onset of puberty and promoting follicular discharge. It is currently known that corticosteroids play a significant role in pregnancy and disease [29], whether they are necessary for the onset of puberty remains unclear. Therefore, further studies need to be conducted to explore how steroid hormones affect the onset of puberty.

In mammals, puberty involves hormone secretion, follicular development, and ovulation; And RNA-seq has been widely used for ovarian transcriptome analysis [17]. Herein 18,139 genes were identified in prepubertal and pubertal goats by RNA-seq, and 75 DEGs met our screening criteria. By KEGG pathway enrichment analysis, 75 DEGs were found to be significantly enriched in Protein digestion and absorption, PI3K-Akt signaling pathway and p53 signaling pathway, which are associated with puberty onset. The PI3K-Akt signaling pathway is closely associated with reproductive development and stimulates mammary gland development during puberty [30]. DNA methylation is known to play an important role in follicle development, and ovarian DNA methylation is significantly enriched in the PI3K-Akt signaling pathway during puberty in pigs [31], so we suggest that this pathway plays an important role in puberty. In addition, p53 is a central mediator that regulates GnRH secretion [32] and stimulate the maturation of the HPO axis and the influence of p53 signaling pathway on puberty cannot be ignored. Network interaction analysis revealed a close relationship among *ZP3*, *HBEGF*, and *THBS1*. The expression of *ZP3* and *HBEGF* was downregulated while that of *THBS1* was upregulated; it is possible that these pivotal genes play a more important role in puberty than others. *HBEGF* affects blastocyst implantation during puberty and transmits signals between the endometrium and trophoblast cells [33], which makes it an important gene in the reproductive process. GO annotation showed that among the terms associated with puberty, such as “reproduction”, “metabolic processes”, and “developmental processes”, *PRLR*, *ZP* (*ZP3* and *ZP4*), *ASTL*, and *THBS1* mRNA were more prevalent. Current research on *PRLR* mRNA mainly focuses on the developmental process of the mammary gland [34, 35]. At the beginning of puberty, *PRLR* expression is upregulated to facilitate the branching of mammary ducts [34]. Ovarian steroid hormones, in particular P_4_, and pituitary hormones, are also essential for the development of the mammary glands [36]. We speculate that the increased expression of *PRLR* stimulates the pituitary and ovary to secrete reproductive hormones: these hormones then partly act on the mammary gland and partly on the ovary to promote ovulation, which in turn drives the development of puberty. *ZP3*, *ZP4*, and *ASTL* expression is downregulated in puberty. Studies on these genes have mainly focused on the prevention of multiple sperm reactions into ovum [37–39]. *ZP* mRNA expression seems to be related to follicle size [40]. In mice, *ZP3* transcripts were found to accumulate until the late stage of oocyte growth [40], and after ovulation, high expression induced acrosome reaction during fertilization [39]. *ASTL*, encoding ovastacin, activates *ZP2* cleavage after fertilization and prevents multifertilization [37]. Puberty means animals attain the ability to reproduce. Together, *ZP3*, *ZP4* and *ASTL* plays an important role in animal fertilization and reproduction control. Meanwhile, the effect of *ZP* mRNA is suspected to promote the development of puberty in goats by indirectly promoting the action of reproductive hormones required for follicular growth on the ovary by affecting follicle size. *THBS1* also participates in promoting ovulation and luteinization [41]; it plays a key role in luteolysis and is influenced by reproductive hormones. Prostaglandin stimulates *THBS1* mRNA expression, which is inhibited by LH and insulin [42]. Furthermore, *THBS1* is reportedly an LH-stimulated, proangiogenic factor during the transformation of the dominant follicle into the young corpus luteum [41]. It has been previously reported that *THBS1* plays a critical role in not only ovulation and corpus luteum formation but also follicular angiogenesis, which is essential for follicular development, excretion and luteinization [41]. On transcriptome analysis, *THBS1* expression was found to be upregulated during puberty, along with a surge in gonadotropins, suggesting that the expression of *THBS1* and changes in gonadotropins are somehow linked and that they together regulate puberty.

## Conclusions

In this study, we first investigated changes in serum gonadotropins and steroid hormones in pubertal and prepubertal goats. Serum FSH, LH, E_2_, Cortisol, Doc, 11-Deo, Cort, and cortisone levels showed an upward trend during puberty, while P_4_ levels showed a downward trend. RNA-seq led to the identification of 75 DEGs (32 up- and 43 downregulated) in pubertal goats. GO and KEGG pathway enrichment analyses revealed that DEGs were involved in, for example, “metabolic process”, “signaling”, “reproduction”, and “growth”. Our results provide a more comprehensive picture of hormonal changes during puberty in goats and point to ovarian DEGs that may be associated with puberty. Our study provides some reference value for exploring the puberty onset in goats from an endocrine and genetic perspective.

## Material and methods

### Animals and sampling

**Fig. 6:**
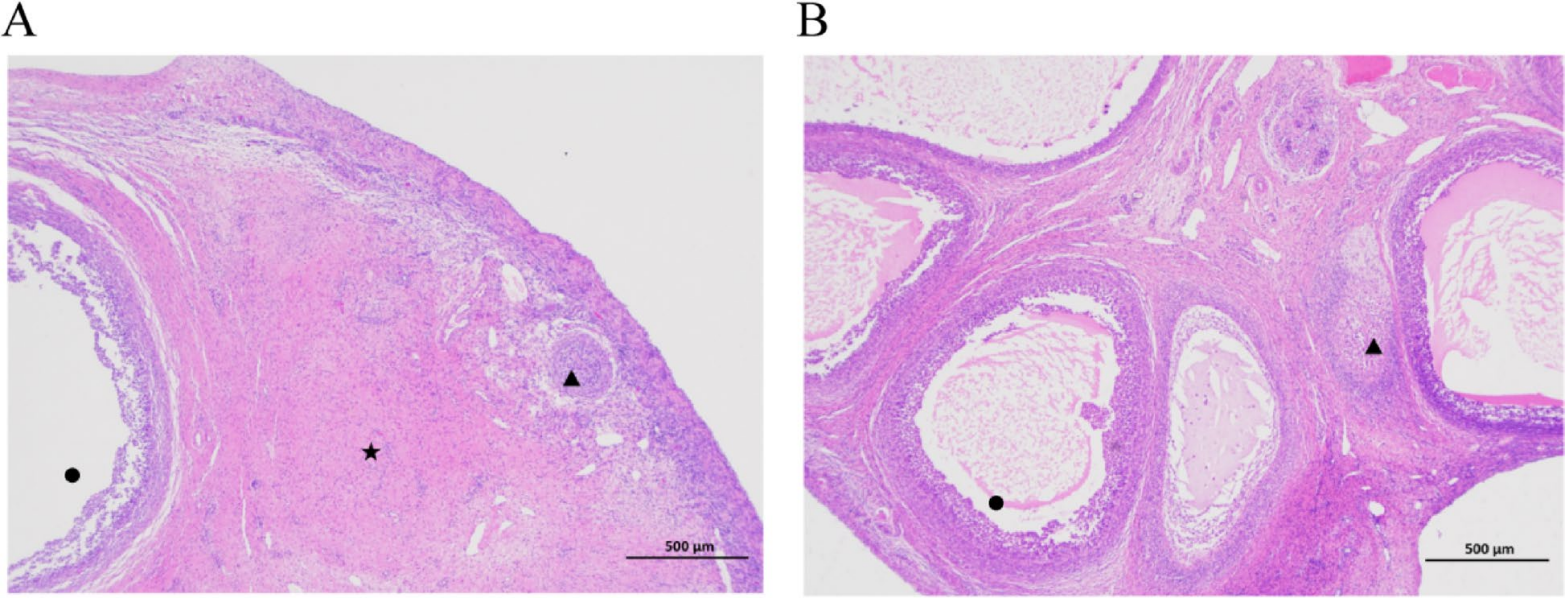
Representative images of pubertal (**A**) and prepubertal (**B**) ovaries. ▲ Secondary follicle; ● Antral follicle; ★ Corpus luteum

We housed 6 AWGs, an indigenous Chinese breed, under the same conditions from BoDa farm, Hefei, Anhui Province, China. All experimental procedures and animals were approved by the Animal Care and Use Committee of Anhui Agricultural University. Prepubertal AWGs (Pre, *n* = 3) were aged 2.5–3 months and weighted 8.1 ± 0.3 kg at ovaries harvested, whereas pubertal AWGs (Pub, *n* = 3) were aged 4.5–5 months and weighted 20.16 ± 0.35 kg at ovaries collected. A detection of individual natural estrus was actualized on 4.5-month-old female goats using a ram and by observing physiological changes in the vulvae and reproductive tract [43]. The age of puberty onset was defined as the age when the female goat accepted the ram to climb across and when the cunnus became inflamed, and meanwhile observed several mature follicles and corpus luteum in ovaries (Fig. 6) [44]. Six blood samples were obtained from the jugular vein of prepubertal as well as pubertal goats at 8:00 a.m before morning feeding. The animals were anesthetized by injecting 0.1 mL/10 kg xylazine hydrochloride (HuaMu China, lot no. 150804) and their ovaries were harvested. Blood (2 mL) stored at 4℃, and then centrifuged at 3000 rpm for 10 min. Serum was separated and stored at −20℃. In order to ensure the accuracy of the results, the right ovaries of goats were collected uniformly. The right ovaries were frozen in liquid nitrogen and stored at − 80℃ until needed for RNA extraction.

### ELISA to assess LH, FSH, E_2_, and P_4_ levels

LH, FSH, E_2_, and P_4_ levels were measured by ELISA [45]. All assays were repeated twice. The following steps were performed in accordance with manufacturer instructions. Serum LH and FSH levels were measured by using kits (KS17416 and KS17435, respectively; Shanghai Keshun Biotechnology Co., Ltd.). The assay sensitivity was < 0.1 mIU/mL, and the coefficient of variation within and between plates was < 15%. Similarly, serum E_2_ and P_4_ levels were measured by using kits (KS17438 and KS17380, respectively; Shanghai Keshun Biotechnology Co., Ltd.). The assay sensitivity was < 0.1 ng/mL and the coefficient of variation was < 15%.

### Ultraperformance liquid chromatography–multiple reaction monitoring–multistage/mass spectrometry (UPLC-MRM-MS/MS) for 11-Deo, Doc, Cort, cortisone, and cortisol

#### Metabolite extraction

Metabolite extraction was performed as previously reported [46]. Briefly, 200 μL sample solution, which was thawed and vortexed for 10 s, was mixed with 20 μL of internal standard solution and 300 μL methanol. The mixture was vortexed for 1 min. Then, 400 μL water was added to the mixture, followed by vortexing for 1 min. After centrifugating for 10 min, the mixture was transferred to a well on the OASIS PRiME HLB µElution Plate (Waters) for solid-phase extraction. Activation was achieved by adding 200 μL methanol and 200 μL water to the plate. After sample loading, the plate was washed with 200 μL of 10% acetonitrile/water (v/v) and 200 μL hexane. Subsequently, 40 μL of 90% acetonitrile/water (v/v) was added, and the eluent was collected. Finally, 60 μL water was added to the eluent. After mixing for 3 min, the supernatant was analyzed by UHPLC-MS–MS.

#### UHPLC-MS/MS and method verification

The analysis was performed on the SCIEX ExionLC™ System (SCIEX, USA). Chromatographic separation was performed on a Waters ACQUITY UPLC BEH C8 column (100 × 2.1 mm, 1.7 μm). The column temperature was set at 50 °C. The temperature of auto-sampler was set at 4 °C and the injection volume was 10 μL. The mobile phases were 0.5 mmol/L NH_4_F in water (A) and methanol (B). Mass analyses were performed on a 6500 QTRAP + triple quadrupole mass spectrometer (SCIEX) [47] equipped with an IonDrive Turbo V electrospray ionization interface. Analytes were monitored by MRM. Table 1 shows optimized quantitative and qualitative changes. Data acquisition and processing were performed using SCIEX Analyst Work Station Software (v1.6.3) and SCIEX MultiQuant™ (v3.0.3), respectively [48].

**Table 1:** Optimized quantitative and qualitative changes in five hormones by multiple reaction monitoring

Abbr.	Prec Ion	Prod Ion	Polarity
Doc	331.2	97.1	Positive
Cort	347.2	121.2	Positive
11-Deo	347.2	109.1	Positive
Cortisone	361.2	121.2	Positive
Cortisol	363.1	115.1	Positive

*Doc* 11-deoxycorticosterone, *11-Deo* 11-deoxycortisol, *Cort* Corticosterone

For all assays, precision was evaluated by calculating the relative standard deviation of repeat measurements of quality control samples. The average recoveries of all target compounds were between 86.8% and 113.4%, and relative standard deviation was < 8.2%. These data indicated that the method was highly reliable.

### RNA-seq

#### Total RNA extraction, cDNA library construction, and sequencing

For each sample, total RNA was extracted from prepubertal and pubertal goat whole ovaries with Trizol (Invitrogen, Carlsbad, CA, USA). Quantification of total RNA was performed using NanoDrop and Agilent 2100 Bioanalyzer (Thermo Fisher Scientific, MA, USA). Subsequently, mRNA purification and cDNA library construction were performed according to manufacturer instructions (MGI, Shenzhen, China). Briefly, total RNA samples were digested by DNase I. mRNA purified with oligo (dT)-attached magnetic beads was sheared into small fragments using a fragmentation buffer at appropriate temperature. Random primers were used for cDNA synthesis, followed by terminal repair and splice connection. After PCR of the conjugated product and digestion of uncyclized linear DNA molecules, the final library was obtained. Library quality was evaluated on Agilent 2100 Bioanalyzer and by RT-qPCR. The quality qualified library was replicated by rolling ring to produce DNA nanospheres, followed by sequencing on the BGI2000 platform (BGI-Shenzhen, China).

### Analysis of RNA-seq data

The raw sequencing data were filtered by SOAPnuke (v1.5.2, BGI-Shenzhen, https://github.com/BGI-flexlab/SOAPnuke) [49] to remove low-quality reads, reads with joint contamination, and reads containing > 5% unknown nucleotides to obtain clean data, which were stored in FASTQ format. Q20, Q30, and GC contents of the clean data were then determined. High-quality clean data were used for all subsequent analyses.

*Capra hircus* genome sequence was downloaded from NCBI (https://www.ncbi.nlm.nih.gov/assembly/GCF_001704415.1#/st). All clean reads were mapped to this genome sequence using HISTA2 (v2.0.4, http://www.ccb.jhu.edu/software/hisat/index.shtml) [50] and then aligned using Bowtie2 (v2.2.5, http://bowtie-bio.sourceforge.net/bowtie2/index.shtml) [51]. Subsequently, the expression level of each sample was determined by RSEM (v1.2.8, https://github.com/deweylab/RSEM) [52]. DEGs were identified based on the negative binomial distribution principle [53]. Transcripts with *Q-*value (corrected *P*) ≤ 0.05 and |log_2_ FC|≥ 1.0 were considered to be differentially expressed. In order to ensure test accuracy, a hierarchical cluster algorithm was applied to DEGs.

### Bioinformatics

GO analysis was performed to determine the main functions of DEGs (http://www.geneontology.org/). Consequently, DEGs were assigned to the following categories: BP, CC, and MF. GO terms with *P* ≤ 0.05 were considered to be significantly enriched. Pathway enrichment analysis was then performed using the KEGG database (https://www.kegg.jp/), and the significance was set at *P* ≤ 0.05. Both GO and KEGG pathway enrichment were analyzed by the Fisher’s exact test. A gene interaction network was constructed by submitting gene symbols to the STRING database (http://string-db.org/) and visualized by Cytoscape (v3.9.1, https://cytoscape.org/). For our analysis, we used a minimum composite score of 0.4.

### RT-qPCR

The expression levels of some DEGs were validated by performing RT-qPCR. Total RNA was extracted from prepubertal (*n* = 3) and pubertal (*n* = 3) goat left ovary tissues using an E.Z.N.A.™ Total RNA Kit II (Omega, USA) and then reverse transcribed into cDNA by an EasyScript® One-Step gDNA removal and cDNA Synthesis SuperMix (TransScript, China), as per manufacturer instructions. cDNA obtained after reverse transcription was tenfold diluted before RT-qPCR. The cycling program was as follows: 95℃ for 30 s and then 40 cycles of 95℃ for 10 s and 60℃ for 30 s, followed by a terminal hold at 4℃. *β-actin* and *GAPDH* served as internal controls. The expression level of each gene was calculated using the 2^−ΔΔCT^method. All primer sequences are listed in Table 2. RT-qPCR analyses were carried out in triplicate from each goat in each group.

**Table 2:** Primer sequences used for RT-qPCR

Gene name	GenBank accession number	Forward primer	Reverse primer	Product length (bp)
*IGF1*	XM_005680539.3	GGATGCTCTCCAGTTCGTGT	TGAGAGGCGCACAGTACATC	161
*SFRP4*	XM_005679147.3	TCCTCATCACCCATCCCTCG	GCGCCACTCGTAACACATGA	111
*DUSP1*	XM_018065720.1	CTGCGAACTGTCCCAACCAT	ACACCCTTCCACCAGCATTC	144
*RCAN1*	XM_018055891.1	GCGATCAGGAGAACGAGGAAGAAG	AGTGGATGGGCGTGTACTCTGG	99
*PADI6*	XM_018054709.1	CTTCGACTGCTACCTGACCG	TGTACCAGACGCTGTCAACC	185
*β-ACTIN*	XM_018039831.1	CACGGTGCCCATCTACGA	CCTTGATGTCACGGACGATTT	158
*GAPDH*	XM_005680968.3	AGGTCGGAGTGAACGGATTC	CCAGCATCACCCCACTTGAT	259

### Statistical analysis

Data processing was performed with Microsoft Excel 2019 (Microsoft, Bellevue, USA) and graphs were generated using GraphPad Prism (version X; La Jolla, CA, USA). Analysis of variance was performed on repeated measures to determine significance, and the significance of the difference between two means was assessed using Student’s *t*-test. Values represent mean ± SEM, unless otherwise stated. *P* < 0.05 indicated statistical significance.

## Supplementary Information

**Additional file 1: Figure S1.** Cluster analyses of DEGs.

**Additional file 2: Table S1.** Reads filter and comparison.

**Additional file 3: Table S2.** Details of DEGs.

**Additional file 4: Table S3.** GO analysis of DEGs.

**Additional file 5: Table S4.** KEGG analysis of DEGs.

## Data Availability

The datasets used and/or analysed during the current study are available from the corresponding author on reasonable request. The sequencing data were submitted to the Sequence Read Archive (Accession Numbers PRJNA944802) in NCBI.

## Abbreviations

AWGs: Anhui white goats
BP: Biological processes
CC: Cellular components
Cort: Corticosterone
DEGs: Differentially expressed genes
Doc: 11-Deoxycorticosterone
E_2_: Estradiol
FC: Fold change
FSH: Follicle stimulating hormone
GO: Gene ontology
HPO: Hypothalamic–pituitary–ovarian
KEGG: Kyoto Encyclopedia of Genes and Genomes
LH: Luteinizing hormone
MF: Molecular functions
RNA-seq: RNA-sequencing
RT-qPCR: Real-time quantitative PCR
11-Deo: 11-Deoxycortisol
