# Supplementary figures and images for: Analysis of serum reproductive hormones and ovarian genes in pubertal female goats
Source: J Ovarian Res. 2023 Apr 6;16:69. doi: 10.1186/s13048-023-01150-0 (PMC10080748; doi:10.1186/s13048-023-01150-0)

差异基因聚类热图(prepuberty-vs-puberty)

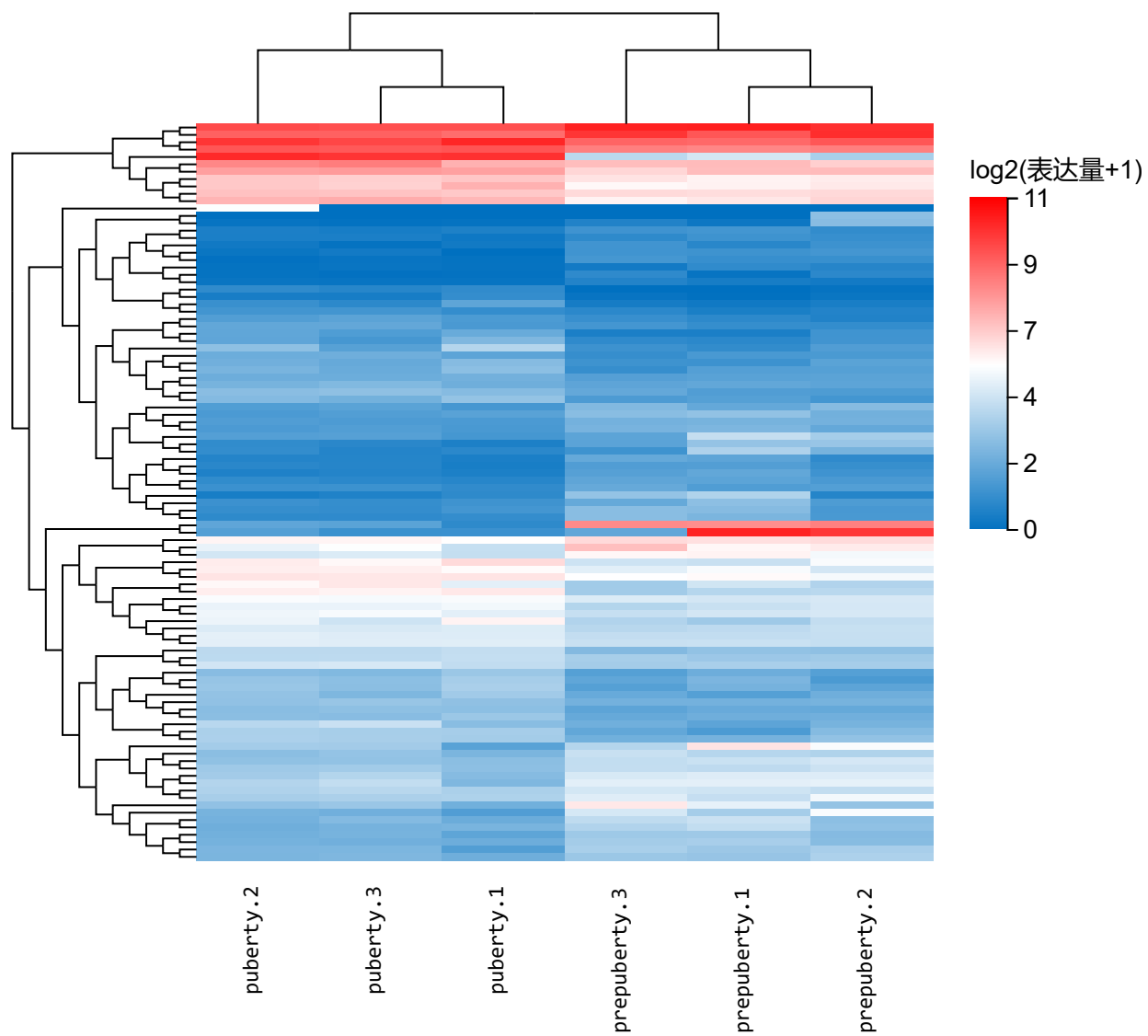

Supplement: Supplementary file 1 — Additional file 1: Figure S1. Cluster analyses of DEGs. [file 13048_2023_1150_MOESM1_ESM.pdf]
